# Identification and Differential Expression of a Candidate Sex Pheromone Receptor in Natural Populations of *Spodoptera litura*

**DOI:** 10.1371/journal.pone.0131407

**Published:** 2015-06-30

**Authors:** Xinda Lin, Qinhui Zhang, Zhongnan Wu, Yongjun Du

**Affiliations:** 1 College of Life Sciences, China Jiliang University, Hangzhou, Zhejiang, China; 2 Institute of Health & Environmental Ecology, Wenzhou Medical University, Wenzhou, Zhejiang, China; Institute of Zoology, Chinese Academy of Sciences, CHINA

## Abstract

Olfaction is primarily mediated by highly specific olfactory receptors (ORs), a subfamily of which are the pheromone receptors that play a key role in sexual communication and can contribute to reproductive isolation. Here we cloned and identified an olfactory receptor, *SlituOR3* (Genbank NO. JN835270), from *Spodoptera litura*, to be the candidate pheromone receptor. It exhibited male-biased expression in the antennae, where they were localized at the base of sensilla trichoidea. Conserved orthologues of these receptors were found amongst known pheromone receptors within the Lepidoptera, and *SlituOR3* were placed amongst a clade of candidate pheromone receptors in a phylogeny tree of insect ORs. *SlituOR3* is required for the EAG responses to both Z9E11-14:OAc and Z9E12-14:OAc *SlituOR3* showed differential expression in *S*. *litura* populations attracted to traps baited with a series of sex pheromone blends composed of different ratios of (9Z,11E)-tetradecadienyl acetate (Z9E11-14:OAc) and (9Z,12E)-tetradecadienyl acetate (Z9E12-14:OAc). The changes in the expression level of *SlitOR3* and antennal responses after *SlitOR3* silencing suggested that *SlitOR3* is required for the sex pheromone signaling. We infer that variation in transcription levels of olfactory receptors may modulate sex pheromone perception in male moths and could affect both of pest control and monitoring efficiency by pheromone application after long time mass trapping with one particular ratio of blend in the field.

## Introduction

The olfactory system of insects to sex pheromone is remarkably sensitive and species-specific and, most notably of moths, has been an invaluable model system for studying fundamental aspects of olfaction[1]. Membrane-bound olfactory receptor proteins (ORs) are the key to olfaction. Two types of ORs, one is the very conserved olfactory co-receptors (ORcos) [2], the other is the conventional odor-specific ORs that have lower sequence homology within and between species[3,4]. Identification of candidate OR genes has most commonly been from genome sequence[5,6], sequence information of cDNA library[7–9], or transcriptome sequence[10,11]. The sex pheromone receptors are crucial for both the survival and adaption [12]. A number of insect sex pheromone receptors have been identified from moths. This type of receptors attracted considerable research interest mainly because they play crucial roles in both reproduction and adaptation. Candidate ORs have been identified in economically important insects, such as *Agrotis ipsilon*[13], *Manduca sexta*[14], *Helicoverpa armigera*[5,15,16] and *Spodoptera littoralis* [17–19]. The function of several ORs has been confirmed using the *Xenopus* oocytes to express the receptors and whole-cell voltage clamping to record the neural activity[20,21]. For example, in the silk moth, *Bombyx mori*, two male-specific ORs responds to bombykol and bombykal [20] were identified, and a *S*. *littoralis* sex pheromone receptor was functionally identified using the heterologous expression in *D*. *melanogaster*[18]. HvOR6 was found to be highly tuned to Z9-14:Ald, while HvOR13, HvOR14 and HvOR16 showed specificity for Z11-16:Ald, Z11-16:OAc and Z11-16:OH, respectively in *Heliothis virescens* [21].

Upon recognition of chemical ordours by ORs, then followed by signaling cascade, a neural perception of the odour in the brain was initiated and may provoke a behavioural response. Therefore the extremely variation of OR genes among individuals[22] can alter odour perception[23,24]. In *D*. *melanogaster*, the nucleotide polymorphism of OR [25] may contribute to individual variation in olfactory behavior[26]. The genetic variation of these receptors also allows the adaption of the population to the changing environments and thus important for the maintenance and evolution of species. To reduce the residue of chemical pesticides, mass trapping or mating disruption by synthetic sex pheromone has been widely used to accurately monitor the pest population [27–31]. The olfactory variation would be critical to the efficacy of the application of insect pheromone in the field. Therefore, the study of sex pheromone receptors in moths is not only of great interest for understanding the olfactory system, but also have important implications for the development of new strategies to manage pest species[32].

*S*. *litura* (Lepidoptera, Noctuidae), also known as tobacco cutworm moth, is one of the most serious agricultural pests feeding on a wide range of economically important crops including cotton, lettuce, corn, tobacco papaya and many others[33]. In the past, the highly conserved *S*. *litura* Orco orthologue has been cloned and was shown to be expressed in both sex and localized at the bases of all categories of olfactory sensilla [34]. Also, some work on the olfactory receptor of *S*. *litura* have been studied [35]. In vivo analyses of the genes involved in sex pheromone detection using knockout or transgenic techniques[18] are crucial to unequivocally determine whether receptor specificity alone is sufficient to explain ORN specificity, or whether additional components are also required[36]. RNA interference has been used in the functional study of olfactory receptor genes [14,37–39]. However, the in vivo functional studies on *S*. *litura* ORs are still lacking. We are interested in understanding the molecular mechanism of pheromone signaling and functional characterization of the sex pheromone receptors in *S*. *litura*, by taking advantage of the transcriptome data of *S*. *litura*(Feng et al., BMC genomics, 2015, in press), we cloned and characterized *SlituOR3*, using bioinformatics and molecular approaches, and examine their expression in each sex. We investigated relationships between sex pheromone responses and the expression of receptor at the transcriptional level in moths attracted to traps with different ratios of sex pheromone components by quantitative real-time PCR(qRT-PCR), as well as the molecular function of *SlituOR3* by combination of RNA interference(RNAi) and electroantennogram(EAG).

## Materials and Methods

### Insects and tissue preparation

*Spodoptera litura* were reared in artificial diet at 25±1°C and with humidity 75±5%, L:D 14:10h [40]. Male and female adult moths were collected daily after emergence and then separated. For qRT-PCR, antennae, heads, legs, thoraces, abdomens, wings and proboscis were dissected, eggs, larvae, pupae, and adults were collected, all samples were stored in liquid Nitrogen.

### Total RNA preparation and cDNA synthesis

TRIzol Reagent (Invitrogen, USA) were used to extract total RNAs from tissues listed above and DNase I (Invitrogen, USA) was used to remove DNA. A UV spectrophotometer (HITACHI) was used to check the quality of total RNA, One microgram of total RNA for each reverse-transcription reaction, oligo(dT)_18_ primer and M-MLV reverse transcriptase were used according to the First-Strand cDNA Synthesis Kit protocol (Fermentas, USA).

### Cloning of *SlituOR3*

To clone the full-length *SlituOR3*, 3’ RACE were performed using the GeneRacer Kit (Invitrogen, USA) according to the manufacturer’s manual. PCR was carried out using PlatinumTaq DNA Polymerase, GeneRacer 5’ Primer or 3’ Primer, and *SlituOR3* gene specific-primers(Table 1). The program used for PCR: 94°C for 2 min; 5 cycles of 94°C for 15 s, 72°C for 2 min; 25 cycles of 94°C for 15 s, 60°C for 30 s, 72°C for 2 min; and one final cycle at 72°C for 5 min. The fragments were then subcloned and sequenced. Base on the sequence result, *SlituOR3* was amplified in a Mastercycler EP Gradient PCR Machine (Eppendorf, Hamburg, Germany), using the following program: 94°C for 2 min; followed by 33 cycles of 94°C for 30 s, 55°C for 35 s, 72°C for 65 s; followed by one cycle at 72°C for 10 min. The PCR reaction consisted of 1μl cDNA, 1 μl each of forward and reverse primers, 12.5 μl, DreamTaq PCR Master Mix (2X), and double-distilled water to a total volume of 25 μl. cDNA fragments were subcloned into the pGEM-T Easy Vector System (Promega, USA) and sequenced by Life technologies Co. (Shanghai, China).

**Table 1:** Primer sequences designed for ORs in RT-PCR, real-time quantitative PCR (RT-qPCR), 3’RACE, dsRNA synthesis, the reference gene SlituRPL8 in RT-qPCR, and the control GFP in dsRNA synthesis.

Application	Gene	Forward primer	Reverse primer
RT-PCR	*SlituOR3*	GGAATTCATGGACACCCTTCAGAAGATAT	CCAAGCTTCTATTGGATAGTAGTCTCTACACCT
qRT-PCR	*SlituOR3*	GCTGCCAAATCGGTTCATAT	AGTCTCCATCGCATGTTCAG
	*SlituRPL8*	ATGCCTGTGGGTGCTATGC	TGCCTCTGTTGCTTGATGGTA
3’RACE	*SlituOR3*	CTCTCTGCGTAATCTGGTTTTGCATGTGT	CTAATACGACTCACTATAGGGCAAGCAGTGGTATCAACGCAGAGT
dsRNA synthesis	*SlituOR3*	TAATACGACTCACTATAGGGAGACCAC TAAACCTGGTGGCACACAAA	TAATACGACTCACTATAGGGAGACCAC GTACACGGCGCTTGGTAACT
	*GFP*	TAATACGACTCACTATAGGGAGATTTGTATAGTTCATCCATGCCATGT	TAATACGACTCACTATAGGGAGAATGAGTAAAGGAGAAGAACTTTTCA

### Sequence analysis

Sequence analyses and homologues searching comparisons were performed using the BLAST (www.ncbi.nlm.nih.gov) program from nucleotide collection (nr/nt)(except Human and Mouse) in GenBank. Sequence alignment was done using CLUSTALW [41]. Phobius (http://www.ebi.ac.uk/Tools/pfa/phobius/) and MEMSAT3 (http://bioinf.cs.ucl.ac.uk/psipred/) were used to predict the trans-membrane domain. A phylogenetic tree was constructed using the Neighbor-Joining method of MEGA5 [42] with a bootstrap of 1,000 replications, totally 56 ORs were used and *SlituOR18* was used as an outgroup

### Gene expression analysis by quantitative real-time PCR (qRT-PCR)

The recipe of the qRT-PCR reaction: 10 μl Ssofast Evagreen (Bio-Rad), 0.75 μl 10μM forward and reverse primers, 1 μl cDNA and 7.5 μl nuclease free water, total volume = 20μl. qRT-PCR was carried out as the following program: an initial cycle at 95°C for 30s, then followed by 39 cycles of 95°C for 5 s, 60°C for 25 s, 72°C for 30 s. Dissociation curves were used to check for the presence of non-specific dsDNA SYBR Green hybrids. The data was analyzed using ABI StepOne Software v2.1 (Applied Biosystems). The expression level of *SlituOR3* was normalized against that of *SlituRPL8*. 2^−ΔΔCT^ method was used where ΔΔC_T_ = (C_T_, SlituOR gene − C_T_, *SlituRPL8* gene) different tissues or stages−(C_T_, SlituOR gene − C_T_, *SlituRPL8* gene) maximum. The experiment was repeated for three times.

### *In situ* hybridization

The fluorescence-labeled RNA hybridization probes used for *in situ* hybridization were synthesized from Life technologies Co. (Shanghai, China): *SlituOR3* (5’-CGCTTGGTAACTTTTCGCTCTCAG-3’) with 5’ Cy3 fluorescence-labeled. Antennae(1- to 2-day-old adult) were embedded in Tissue-Tek OCT Compound (Sakura, Japan) and frozen at -20°C. Cryosections (7μm) of antennae were thaw-mounted on Superfrost Plus slides (Fisherbrand, USA) and air-dried at room temperature for 30 min. Slides were then treated at 4°C with 4% paraformaldehyde in PBS (phosphate-buffered saline: 0.85% NaCl, 1.4 mM KH_2_PO4, 8 mM Na_2_HPO4, pH 7.1) for 30 min, 1×PBS for 2×5 min, 0.2 M HCl for 8 min, 1×PBS for 2×5 min, 1×PBS with 1%Triton X-100 (Amresco, USA) for 10 min followed by a 5 min washes in 1× PBS. Finally, slides were rinsed(10 min) in 50% formamide, 5 × concentrated SSC (1× SSC = 0.15 M NaCl, 0.015 M Na-citrate, pH 7.0) and drained. Then sections were covered with 100 μl hybridization solution (50% formamide, 2× SSC, 10% dextran sulphate, 20 μg/ml yeast t-RNA, 0.2 mg/ml herring sperm DNA) containing a fluorescence-labeled antisense RNA (levels:0.5–1μg/ml). The samples were then covered with a coverslip and slides were incubated in hybridization instrument (StatSpin TermoBrite, USA) at 55°C overnight. Post-hybridization were washed 3 times for 5 min in 2 × SSC at 37°C, then washed three times for 5 min in 0.2 × SSC at 37°C, then three times for 5 min in 1 × TBS (Tris-buffered saline; 100 mM Tris, pH 7.5, 150 mM NaCl). Time of washing was determined by observing under the fluorescence microscope. Samples were mounted using the DAPI/Antifade Solution (Chemicon, USA). Images were acquired on a Nikon SiA1 laser confocal fluorescence microscopy (Japan).

### Variation of receptor expression and responses to sex pheromone in field-trapped populations

To test whether the transcriptional level of OR expression is related to differential behavioral responses to pheromone mixtures, we baited moth traps with different ratios of two *S*. *litura* sex pheromone components. *S*. *litura* Males attracted were collected and the expression of *SlituOR3* was measured using qRT-PCR.

### Pheromone lures

The two *S*. *litura* sex pheromone components (9Z,11E)-tetradecadienyl acetate (Z9E11-14:OAc) and (9Z,12E)-tetradecadienyl acetate (Z9E12-14:OAc) (Bedoukian Research, Inc., Danbury, USA) were purified by flash column chromatography (silica gel impregnated with 15% silver nitrate) and the purity of each was shown by gas chromatography to be >95%. Moth pheromone traps were baited with lures containing Z9E11-14:OAc and Z9E12-14:OAc were presented in specific blends. Eight mixtures of the two pheromone components were prepared in ratios of Z9E11-14:OAc: Z9E12-14:OAc ranging from 1:2 to 12:1. To maintain the release rate, the mixtures were diluted to the desired concentration in corn oil and injected into PVC capillary tubing (ca. 80 mm length, id 0.6 mm and od 1.1 mm) (NewCon Inc., Ningbo, China), the ends of which were then heat sealed to form the pheromone lure. Lures were sealed in aluminum foil bags, stored in -20ଌ refrigerator and shipped by courier to test locations when needed.

### Trapping of moths

Plastic noctuid moth traps (NewCon Inc., Ningbo, China) were deployed and set up at a height of about 1 m in the Longwan vegetable field in Wenzhou, Zhejiang (120°82'E, 27°93'N). No special permits were required for field collection and sample processing. Collection permission was obtained from the land owners. The field studies did not involve endangered or protected species. They were distributed equidistant from each other at a density of 15 traps per ha. Each trap was baited with a pheromone lure or was un-baited as a control trap (CK), treatments being allocated at randomly. The experiment had 6 replicates for each treatment. Trapped moths were collected and counted daily. Moth antennae were dissected and immediately placed into liquid nitrogen. *S*. *litura* males were attracted by pheromone mixtures and antennae of twenty males were collected for each mixture. Total RNAs were then prepared, and transcribed into cDNA. qRT-PCR measurement of the expression of *SlituOR3* was done as described above and the expression levels were compared among different Z9E11-14:OAc and Z9E12-14:OAc ratios of pheromone lure. For comparison, the expression of SlituORco, was also tested. At least 3 replicates were made for each treatment.

### RNA interference

The fragment of *SlituOR3* were amplified by PCR and used for dsRNA synthesis. For GFP (Green Fluorescent Protein) dsRNA synthesis, a fragment was amplified by PCR using pMD18T-GFP as a template kept in the lab. All the primers used were listed in Table 1. dsRNA was synthesized using an Ambion MEGAscript RNAi Kit and transcription performed following the manufacturer’s protocol (www.ambion.com). For the injection, dsRNA was diluted in injection buffer (0.1 mM sodium phosphate, pH 6.8; 5 mM KCl) in concentrations of 1.0 μg/μl.

The pupa was positioned with fingertips so that the abdomen can be approached with the injecting bevel-tip micro-syringe (Agilent, USA). A volume of 0.2 l pupa-1 was injected into the abdomen. Typically, the injection site was in the ventral mid-lateral part of the abdomen at the level between the 3rd and the 4th sternite.

### Recording of EAG responses

Two *S*. *litura* sex pheromone components Z9E11-14:OAc, Z9E12-14:OAc and one plant volatile component (3Z)-hexenyl acetate (Z3-6:OAc) (Aladdin reagent Inc., Beijing, China), were diluted in liquid paraffin to give 100μg/μl solution(10^−2^). A piece of filter paper (8x30 mm) impregnated with 20μl of test solution was inserted into a glass Pasteur pipette after the solvent evaporated and used as a stimulus cartridge. The cartridge was freshly made each time and the end was sealed with Parafilm until use. The EAG signals were recorded and analyzed by Syntech system (Syntech, The Netherlands)[43]. The antenna was cut from male moth post eclosion, and an electroconductive gel (World Precision Instruments Inc., USA) was used for the maintenance of electrical contact between the antenna and the electrodes. The stimulus parameters are: 50 ml/min the clean airflow, 0.1s the stimulus time, and 60 s the interval time. The recording data was analyzed from the antennae of three male moths.

### Statistical analysis

Statistical analysis was conducted using SAS 9.2. One-way ANOVA and by two-way ANOVA were used to analyze the differences between pheromone component ratios, the numbers of moths caught and the expression of *SlituOR3*. Student’s t test were used for comparison of *SlituOR3* expression levels and EAG responses between *SlituOR3* dsRNA silencing and control(dsGFP). Duncan’s multiple range tests were used for multiple comparisons of the expression levels of *SlituOR3* in different tissues and antennae of both sexes of *S*. *litura*, expression levels during the development of *S*. *litura*, the expression in antennae of moths trapped by different ratios of Z9E11-14:OAc and Z9E12-14:OAc, and the expression in the antennae of male *S*. *litura* post eclosion after dsRNA injection.

## Results

### Cloning of *SlituOR3*

**Fig 1:**
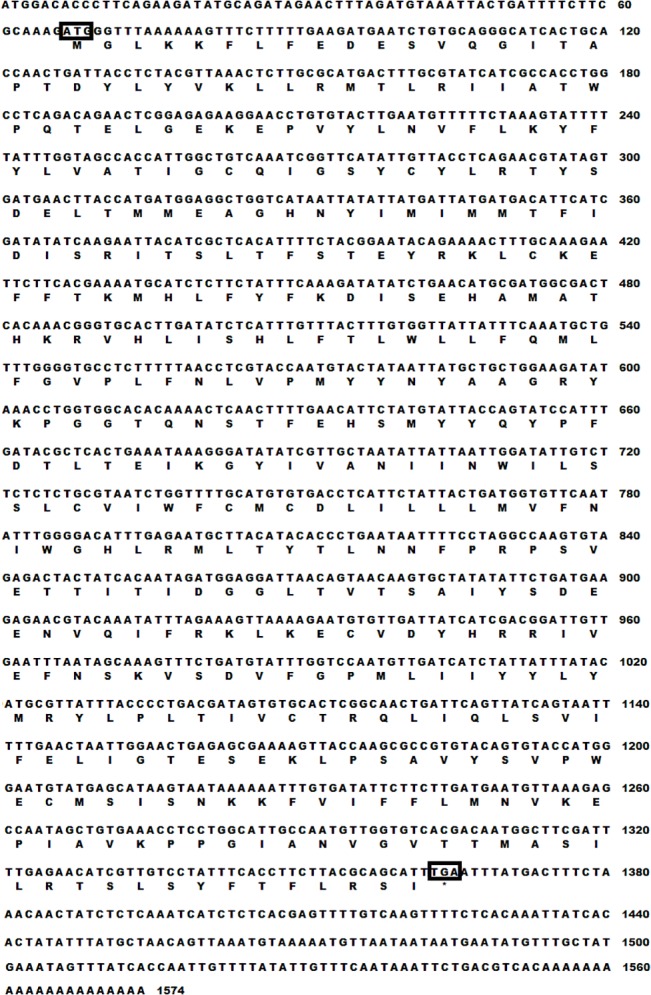
Nucleotide sequence encoding the *SlituOR3* gene of *S. litura.* The nucleotides are numbered on the right. The start (ATG) and stop (TGA) codons are boxed. Amino acid sequence below the nucleotide sequence are shown also.

**Fig 2:**
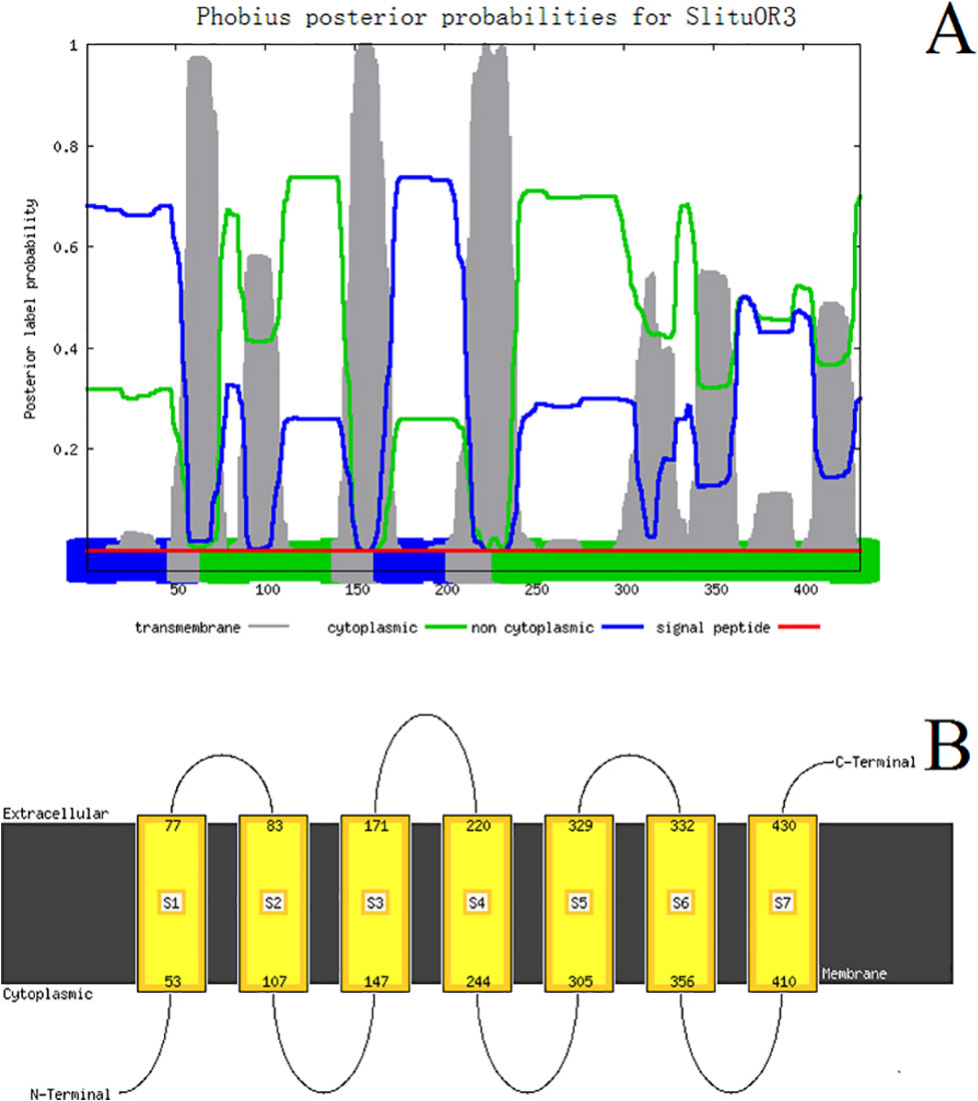
The transmembrane protein topology prediction for *SlituOR3*. A: Prediction using Phobius. The bar beneath the red line shows the predicted results and the plot above provides complimentary information in the form of probabilities. Gray = transmembrane domain, green = cytoplasmic region and blue = extracellular region. The x axis represents the site of the amino acids and the y axis the probability that the amino acids at that site occupy each region or domain. For further information see http://phobius.sbc.su.se/instructions.html. B: Prediction using MEMSAT3. The brown bar = cellular membrane, regions above and beneath the brown bar are the extracellular and cytoplasmic regions respectively; yellow blocks represent transmembrane domains numbered S1-S7 and the numbers at the top and bottom of each yellow block indicate the positions of amino acid residues at each end of the domain. MEMSAT3 predicts the N-termini of *SlituOR3* are cytoplasmic and the C-termini of *SlituOR3* are extracellular. For further information see http://bioinf.cs.ucl.ac.uk/psipred/.

**Fig 3:**
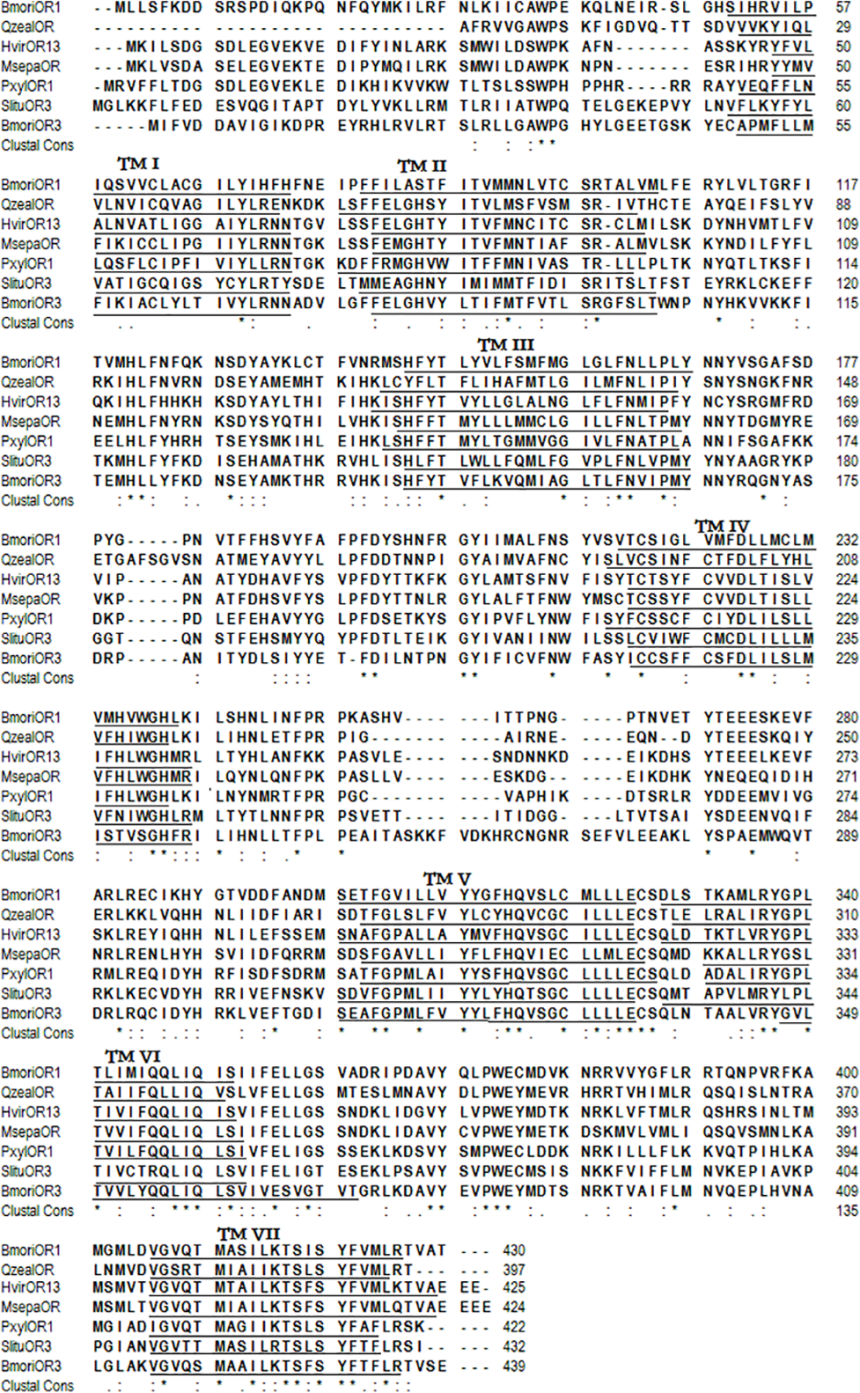
Alignment of amino acid sequences of *SlituOR3* with six verified moth sex pheromone receptors. Gaps are indicated with slash (-). Identical amino acids are marked in the bottom with *. Transmembrane domains identified with MEMSAT3 & MEMSAT-SVM are underlined and numbered I to VII. GI numbers of each OR are: *BmorOR1*(GI:112983558); *BmorOR3* (GI:112982950); *HvirOR13*(GI:51127338); *MsepOR*(GI:226001155), *OzeaOR*(GI:284010026); *PxylOR1*(GI:205361602); *SlituOR3*(GI: 381211953).

Distinct and separate bands were obtained for *SlituOR3* by RT-PCR of antennal cDNA and it was of the expected sizes given the primer design. The *SlituOR3* full-length cDNA sequence was 1574 bp, encoding 432 amino acids (Fig 1). Predicted by the Phobius and MANSAT3, the *SlituOR3* has seven transmembrane domains (Fig 2). Aligning the *SlituOR3* with verified moth pheromone receptors indicated a high degree of conservation across species (Fig 3). *SlituOR3* annotations have been submitted to GenBank and accession numbers is JN835270.

**Fig 4:**
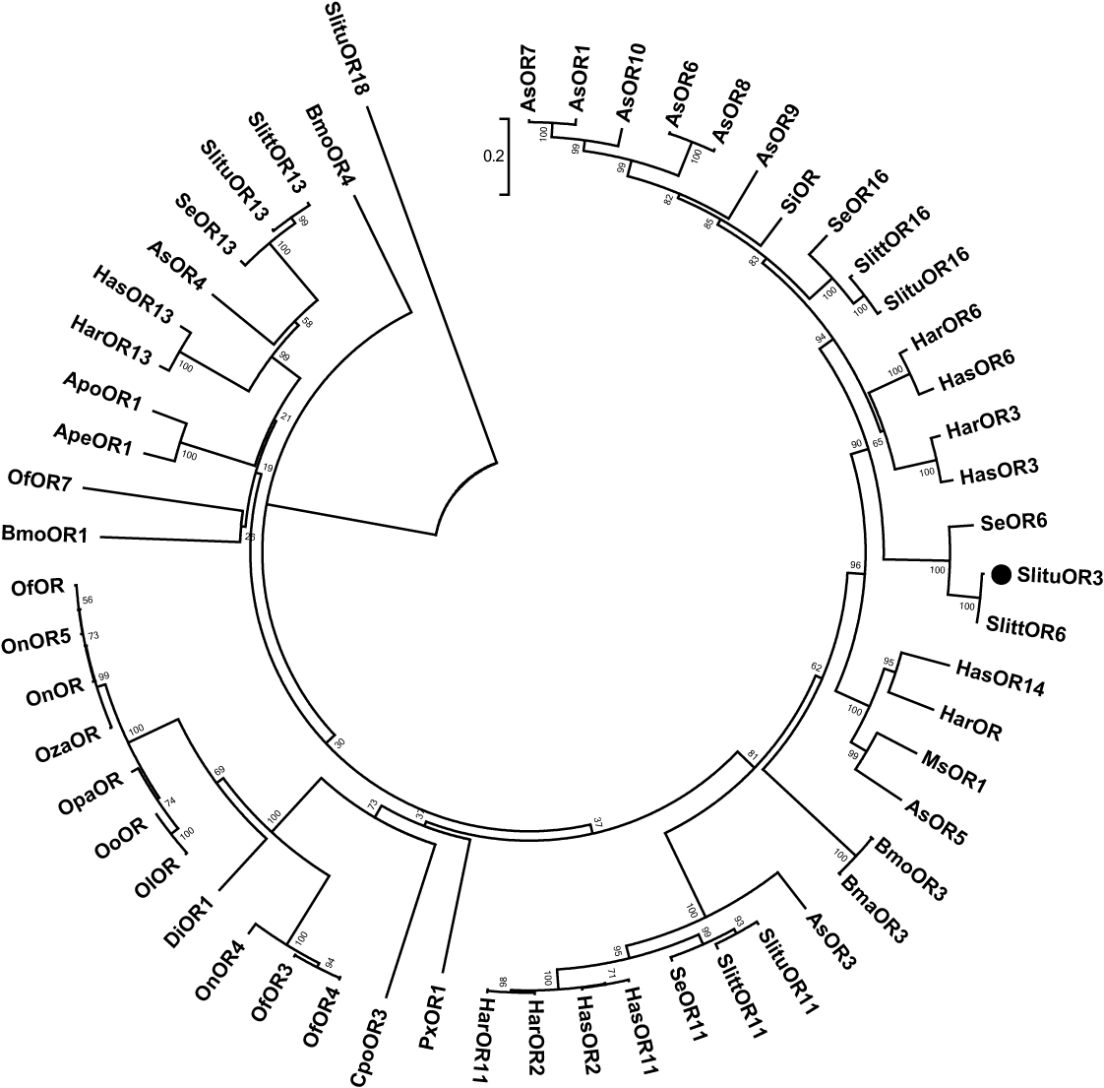
Phylogenic analysis of *SlituOR3* and homologues. Neighbor-Joining method was used. Shown here is the optimal tree with the sum of branch length = 6.00509761. The percentage of replicate trees in which the associated taxa clustered together in the bootstrap test (1000 replicates) are shown also. Poisson correction method was used to calculate the evolutionary distances. CSPR stands for the candidate sex pheromone receptor. *SlituOR18* was used as outgroup. Slitu: *Spodoptera litura* (OR3:AEY84943.2; OR6:AGI96748.1;OR16:AGI96751.1;OR11: AGI96749.1;OR13:ACL81181.1;OR18:AGA16498.1); Slitt: *Spodoptera littoralis* (OR6: ACL81183.1;OR16: ACL81182.1;OR11:ACL81180.1;OR13: AGI96750.1) Se: *Spodoptera exigua* (OR6:AGH58119.1;OR16:AGH58122.1;OR11:AGH58120.1;OR13:AGH58121.1); Si: *Sesamia inferens* (OR: AGY14579.2); Har: *Helicoverpa armigera* (OR6: AGK90000.1;OR3:ACS45306.1;OR:AIG51863.1;OR2: ACS45305.1;OR11:ACF32965.1;OR13:ACJ12370.1);Has: *Helicoverpa assulta* (OR6:AGK90014.1;OR3:ACS45309.1;OR14:AHI44516.1;OR11:AJD81549.1;OR2:ACS45308.1;OR13:AJD81551.1); As: *Agrotis segetum* (OR10:AGS41449.1;OR7:AGS41446.1;OR1:AGS41441.1;OR6:AGS41445.1;OR8:AGS41447.1;OR9:AGS41448.1;OR5:AGS41444.1;OR3:AGS41442.1;OR4:AGS41443.1);Ms:*Mythimna separate*(OR1: BAG71414.1);Bmo:*Bombyx mori* (OR3:NP_001036925.1;OR4:BAH57981.1;OR1:NP_001036875.1);Bma: *Bombyx mandarina*(OR3:ACT34882.1); Px:*Plutella xylostella*(OR1:*AGK43824*.*1*); Ape: *Antheraea pernyi*(OR1: CBH19583.1); Opa:*Ostrinia palustralis*(OR: BAH57978.1);On: *Ostrinia nubilalis*(OR: BAJ61929.1;OR5:ADB89182.1;OR4:ADB89181.1); Oza:*Ostrinia zaguliaevi*(OR: BAH57976.1); Ol:*Ostrinia latipennis*(OR:BAH57981.1); Oo:*Ostrinia ovalipennis*(OR:BAH57979.1);Di:*Diaphania indica*(OR1: BAG71417.1); Of: *Ostrinia furnacalis*(OR:AGG91642.1;OR4:AFK30397.1;OR3:BAR43446.1;OR7:BAR43449.1);Cpo:*Cydia pomonella*(OR3: AFC91713.2);Apo: *Antheraea polyphemus*(OR1: CBH19582.1).

Phylogenetic analysis showed that *SlituOR3* clustered with the ORs containing most closely sex pheromone receptor *S*. *littoralis* OR6 [44] and is related closely to male-specific receptor *H*. *virescens* OR16[45]. And there were so many other male-specific receptors or sex pheromone receptors closely in the cluster: for example, *H*. *virescens* OR14 [45], *H*. *virescens* OR15 [45], *M*.*separate* OR1[46], *M*.*sexta* OR1[47], *H*. *virescens* OR11 [45] and so on. We named this cluster the ‘candidate sex pheromone receptor subfamily (Fig 4).

### Spatial and temporal expression of *SlituOR3*

**Fig 5:**
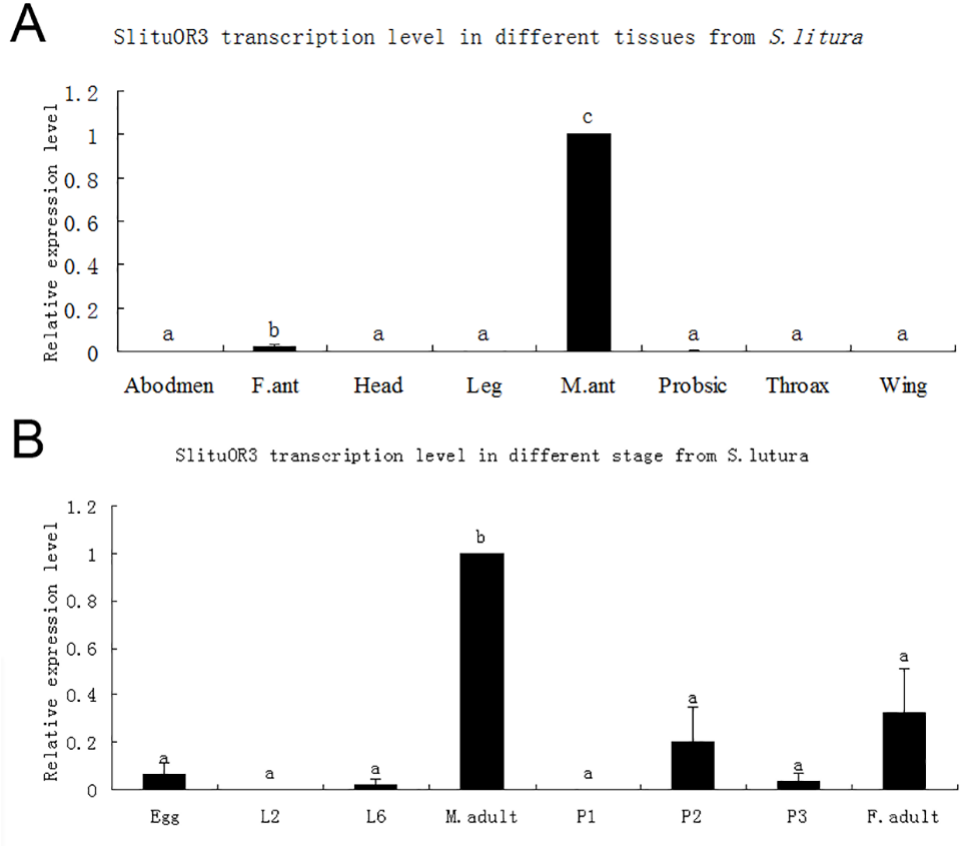
Spatial and temporal expression of *SlituOR3*. A: Expression levels in different tissues and antennae of both sexes of *S*. *litura*. B: Expression levels during the development of *S*. *litura*. Total RNA extracted from all tissues tested were mixed-sex unless otherwise stated. Expression levels were calculated by 2^-ΔΔCt^ method using *SlituRPL8* as the reference gene. Error bars represent standard error. Duncan’s multiple-range test was used, P<0.05. M. ant = antennae of adult male; F. ant = antennae of adult female; E = eggs; L3 = third instar larvae; L6 = sixth instar larvae; P1 = early pupae (1^st^ -3^rd^ day); P2 = mid-stage pupae(4^th^ -5^th^ day); P3 = late pupae(6^th^ -7^th^ day).

The *SlituOR3* showed sexually dimorphic expression. *SlituOR3* was almost exclusively expressed in male antennae (Fig 5A). *SlituOR3* was slightly expressed in tissues including female antennae, proboscis, abdomen and negligible in the head, leg, thorax and wings (Fig 5A). *SlituOR3* expressed mainly in the adult (Fig 5B). Expression of *SlituOR3* was undetectable during larval and early to mid-pupal stages.

### Location of *SlituOR3* in olfactory sensillum

**Fig 6:**
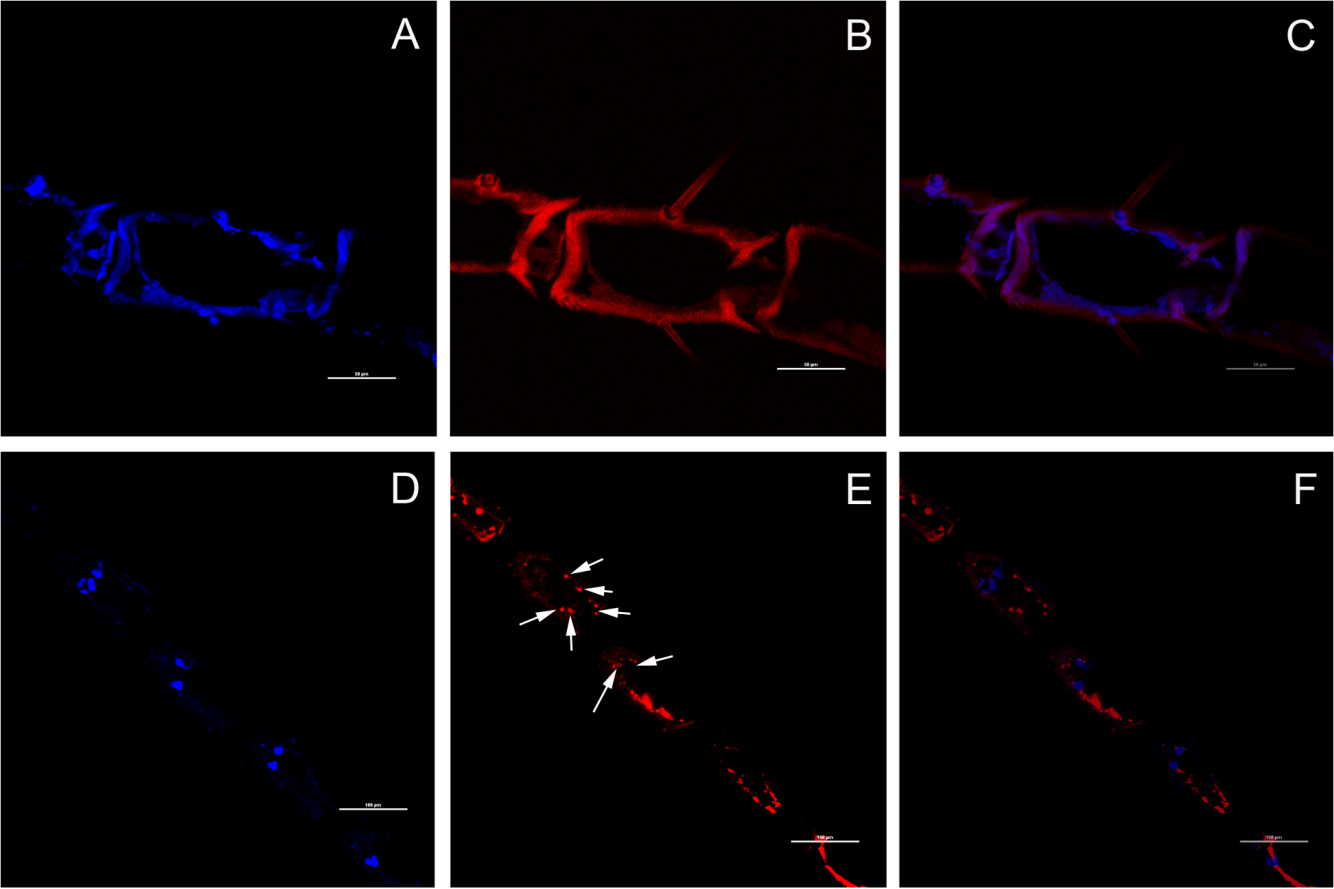
Expression of *SlituOR3* in adult male antennae as visualized using fluorescence-labeled in situ hybridization(FISH). Anti-sense(E,F) and sense(B,C) probe were used.(A-F) Longitudinal sections of hybridized adult male antennae:(A-F)hybridization solution containing fluorescence-labeled probes. A, D: Positive nucleus labeled by DAPI; B, Negative control using a sense probe; E: Positive(anti-sense) *SlituOR3* RNA dyed by Cy3; C: Merge of figuration B and C; F: Merging of figuration D and E. Hybridization signals are indicated with arrows.

The fluorescence-labeled *in situ* hybridization (FISH) result showed that *SlituOR3*-positive RNA was clearly observed in the cryosection and mainly localized at the base of sensilla trichoidea (Fig 6D–6F). The negative control(sense probe) only showed background(Fig 6A–6C). Fig 6E and 6F shows the *SlituOR3*-expressing positive in red fluorescence near the cell nucleus(Blue) but these two signals are not overlapping, indicating that *Slitu*OR3 is not expressed inside the nucleus.

### Differential *SlituOR3* expression and sex pheromone responses in a field population

**Fig 7:**
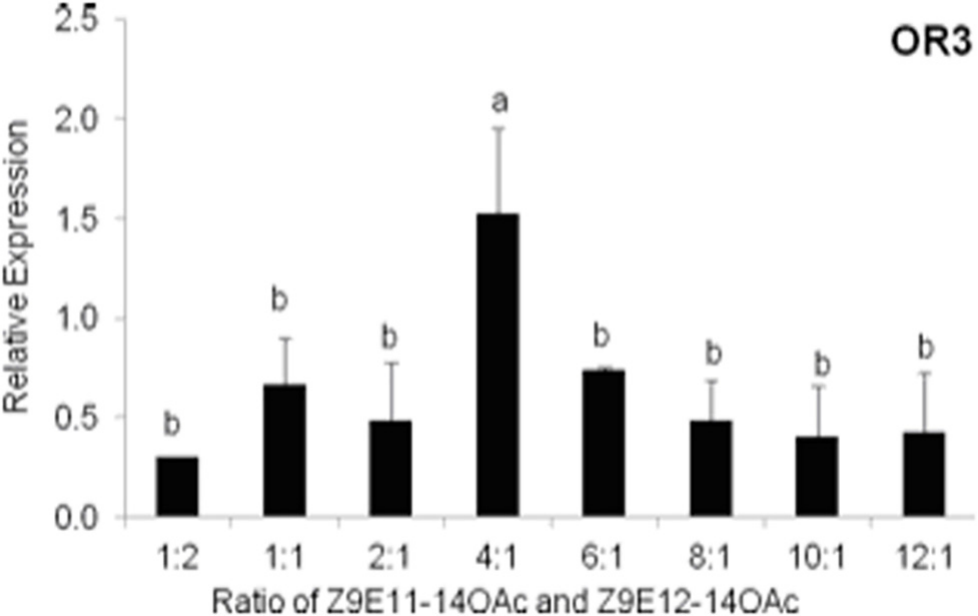
Relative expression of *SlituOR3* and the conserved ORco gene in antennae of male *S*. *litura* caught in traps baited with different ratios of the conspecific sex pheromone components Z9E11-14:OAc and Z9E12-14:OAc in a tobacco field in Wenzhou, Zhejiang. Expression levels are given relative to the expression of the reference gene *SlituRPL8*. Duncan’s multiple-range test was used, P<0.05.

We measured the *SlituOR3* expression in moths collected from the field population to pheromone-baited traps using qRT-PCR. Expression analysis in male moths attracted revealed that *SlituOR3* was differentially expressed in moths attracted by different ratios of the pheromone component (Fig 7). Indeed we also observed some differences in expression of the conserved ORco but its expression levels were more consistent than that of *SlituOR3*. For *SlituOR3* the highest transcription level was in moths trapped by the 4:1 ratio blends of Z9E11-14:OAc and Z9E12-14:OAc, while there was least expression in those caught in traps with a 10:1 blend (Fig 7).

### Silencing of *SlituOR3* reduced the EAG response of male moth

**Fig 8:**
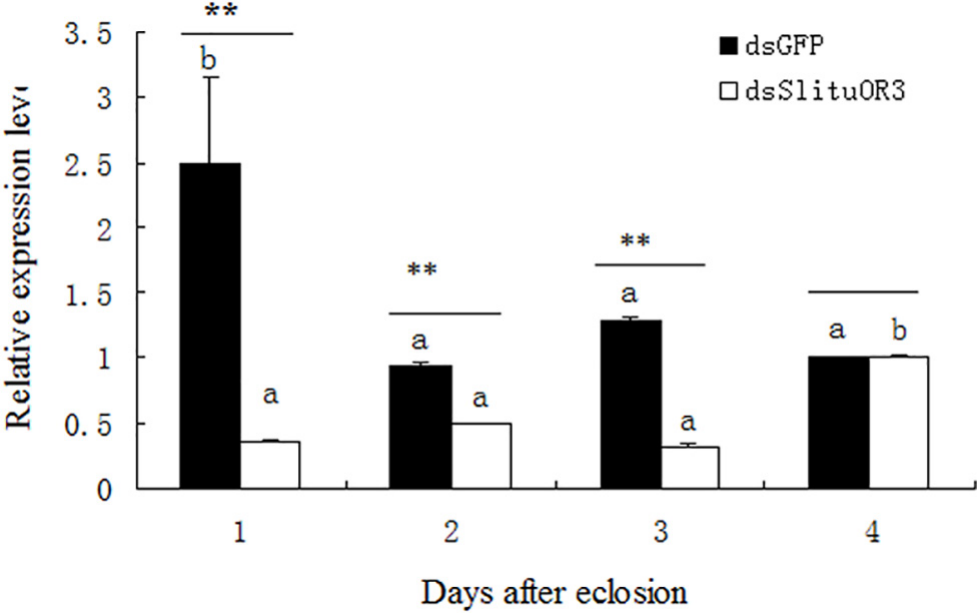
Relative expression of *SlituOR3* in the antennae of male *S*. *litura* post eclosion after dsRNA injection. *SlituRPL8* was used as a reference gene and all expression levels are given relative to the reference gene. t-test and Duncan’s multiple-range test were used(P<0,05, P<0.05).

**Fig 9:**
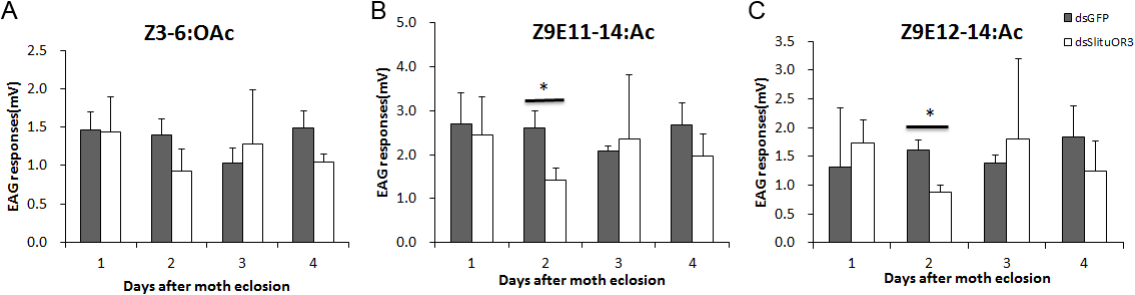
The EAG response recording of male moth antennae with the sex pheromone components Z3-6:OAc(A), Z9E11-14:OAc(B) and Z9E12-14:OAc(C) after injection of *SlituOR3* dsRNA.

We then ask whether *SlituOR3* mediated the response of *S*. *litura* to sex pheromone[37,48]. We silenced the *SlituOR3* by dsRNA in the stage of pupae. Quantitative real-time PCR (QRT-PCR) showed that the expression of *SlituOR3* are significantly reduced in the antennae of male *S*. *litura* post eclosion compared to the control (dsGFP) in the 1, 2, 3 days(Fig 8, P<0.01, P<0.01, P<0.01). This difference disappeared in the fourth day (Fig 8). Then we used Electroantennogram (EAG) to test whether their olfactory responses to pheromones or related molecules were changed. The EAG responses of *SlituOR3* and GFP(control) dsRNA injected males to Z3-6:OAc, Z9E11-14:OAc and Z9E12-14:OAc separately were recorded. The result showed that the EAG responses of the male moths with *SlituOR3* silenced to either Z9E11-14:OAc or Z9E12-14:OAc were significantly low compared to the dsGFP control 2 days after eclosion(P<0.05), while which have not changed significantly to green leaf volatile Z3-6:OAc (Fig 9).

## Discussion

We have cloned and characterized an olfactory receptor from *S*. *litura*, *SlituOR3*. *SlituOR3* was clustered with those functionally-identified sex pheromone receptors of *Bombyx*, *Heliothis*, *Plutella*, *Mythimna*, *Manduca* and *Diaphania* (Fig 3)[8,20,21,49–53]. The high expression in male antennae and our further RNA silencing result suggested that *SlitOR3* is related to the sex pheromone signaling of *S*. *litura* male olfactory system. This result was also confirmed by heterologous expression result in *Xenopus* oocytes, which revealed that *SlituOR6* (equal to our *SlituOR3*) in *S*. *litura* was equally tuned to Z9,E12-14:OAc and Z9-14:OAc, with a small response to the major pheromone component Z9,E11-14:OAc [54].

Natural olfactory stimuli are often complex and highly variable[55]. The quantity and quality of pheromone released from female moths can be affected by host plants [56], diurnal or circadian rhythms[57–59], age[60] and season [61]. Intraspecific divergence in pheromone chemistry has been reported in *Ostrinia nubilalis* [62], *O*. *furnacalis* [63], *Dioryctria abietivorella* [64], *Hemileuca electra*, *H*. *eglanterina* [65,66], *Helicoverpa armigera* [67], and *Agrotis segetum* [68]. The optimal ratio of insect sex pheromone components can be varied by the geographic locations and also by their host plants[69]. Insects perceive and discriminate among such a vast array of sensory cues in their environment. The tobacco cutworm larva is polyphagous[70] and their adult moths are migratory[71]. In the long time of co-evolution, insects adapted to the variation of chemical information from the environment. *S*. *litura* male moths differentially showed attractive to highly variable ratios of Z9E11-14:Ac/Z9E12-14:Ac in field trapping, which has similarly been reported in many other insects, such as *Phyllonorycter ringoniella*[72] and *O*. *furnacalis* males [73], and even in wind-tunnel experiments[74]. In the pheromone mediated mating behavior, the information of sex pheromones was delivered to the opposite sex by the sex pheromone receptors, and these receptors play a crucial role in the chemically mediated mating behavior. Accordingly, the male moths have adapted to the variation of pheromone composition by the variation of their olfactory receptors. Both genetic and environmental factors contribute to individual variation in behavioral responses to these cues[25]. OR genes are extremely variable between individuals[22]. For example, the corn- and the rice-strain of *S*. *frugiperda* are genetically and behaviorally different, which seem to be in the process of sympatric speciation [75]. The polymorphisms in olfactory receptors in *D*. *melanogaster* were identified to be significantly associated with variation in their responses to fruit odorants[25]. The sex pheromone of *S*. *litura* consists of Z9E11-14:OAc and Z9E12-14:OAc[76,77] with the ratio of Z9E11-14:OAc: Z9E12-14:OAc = 100:27 in the pheromone gland [77]. Field-trapping experiments showed that individual variation in behavioral responses of male *S*. *litura* to different ratio of pheromone blends. We speculate that *SlituOR3* is a sex pheromone receptor and mediate these differences in male behavior. *SlituOR3* expressed differentially in the moths attracted by the mixtures constructed from different ratios of Z9E11-14:OAc and Z9E12-14:OAc. Moreover, *SlituOR3* was most abundantly expressed in moths attracted with a 160 μg:40 μg of Z9E11-14:OAc and Z9E12-14:OAc blend (ratio of 4:1), which attracted the largest number of moths at the dose of 200 μg. The minor component Z9E12-14:OAc, which *SlituOR3* responds, plays a key role in the olfactory variation of *S*. *litura* sex pheromone. This variable expression level of *SlitOR3* might be a result of complex transcriptional regulation cascade in response to environmental changes, or other factors mentioned above. Therefore the gene expression difference by the innate transcriptional regulation cascade might be an indicator of genetic variation.

Variability within sex pheromone signaling systems is generally believed to be low because their role in reproductive isolation maintains niche adaptation and leads to strong stabilizing selection. ORs are less conserved and usually specific to odorants than olfactory co-receptors (ORcos) of the OR83b family of proteins [21,37,78,79]. It is likely to be an adaptive advantage that an insect’s system of sex pheromone communication should have some inherent flexibility. There is now evidence that this variability may extend to the intraspecific level. It has been shown that responses to sex pheromones in insects can be modulated by odor experience [80] and that environmental factors may contribute to variation in the pheromone sensitivity of male moth populations [81]. A diurnal rhythm has been observed in the pheromone mediated behavioral activity of field populations of male *S*. *litura* [82]. The reproductive isolation and formation of the new species might be partly contributed by the interaction of sex pheromones and their receptors.

Changes in the pheromone components by the female, lead to reduction of the communication efficiency and cause fitness loss. Therefore broader pheromone components responsive may provide a mechanism for variation in the male moth response that enables population level shifts in pheromone blend use. However, such variation is critical to our application of mass trapping. Only one optimal ratio of pheromone components was formulated as a commercial lure for mass trapping in the pheromone application. Long-term trapping the population with the mixture of such a particular ratio of sex pheromone blend leads to a decline in the proportion of population that responds to such ratio in the field. Eventually, it would cause the shift of optimal pheromone blends of this species and significantly affect the control efficiency and monitoring accuracy when continuously using the commercial pheromone lures in the field. Using voltage clamp electrophysiology, candidate sex pheromone receptors are expressed in *Xenopus* oocytes and receptors highly selective for sex pheromone component or with more broad responses were identified [54,83,84]. However, to answer the question how sex pheromone receptors adapted to different pheromone components, such methods have obvious shortages, ie, the information of the interaction or the feedback role of different ORs in mediating the sex pheromone signaling are usually missing. Moreover, whether the change of male responses was caused by the adaption or by genetic change could not be discriminated. Through RNAi, based on its EAG responses to Z9E11-14:OAc and Z9E12-14:OAc, we surmise that the differential expression of sex pheromone OR that we have shown in *S*. *litura* males attracted to different pheromone blends supports previous studies[54,83,85]. However, the study using heterologous expression in *Xenopus* oocytes showed that *SlituOR3* is tuned to Z9E12-14:OAc but not to Z9E11-14:OAc[54], which might be due to the difference of the heterologous expression and *in vivo* gene silencing by dsRNA. Furthermore, the process of neural signaling by pheromone is much more complicated in measuring the EAG response in the whole antenna than recording of the responses of *Xenopus* oocytes. The functions of ORs in mammalian olfactory system have reported to be modulated by M3 Muscarinic Acetylcholine Receptor[86], thus, the recognition of Z9E11-14:OAc by *SlituOR3* could be dependent on the activity of other receptors, which were not expressed in the heterologous *Xenopus* oocytes. On the other hand, the dosage of sex pheromone stimulants could be another factor affecting the olfactory response of the moth antenna, i.e., in vivo system it is more accessible for the relatively higher dose of pheromone compounds and more sensitive. qRT-PCR results showed that the expression of *slituOR3* significantly decreased in the first three days, while the EAG responses to Z9E11-14:OAc and Z9E12-14:OAc only decreased at the second day. This is possibly due to the complexity of EAG response, and the change of EAG response through gene silencing was related to complicated processes, which delayed the antennal responses to Z9E11-14:OAc and Z9E12-14:OAc. Also, a feedback control after silencing of *slituOR3* terminated the neural responses to Z9E11-14:OAc and Z9E12-14:OAc earlier, while the transcriptional level of *SlituOR3* remains significantly low.

If the significance of sex pheromone communication to adaptation in moths is to be fully understood, there is a need for further studies on the population genetics of both pheromone production and reception and on the regulation and expression of genes critical to sex pheromone communication in both males and females. It is possible that multiple pheromone receptors may be involved in identifying each component. In summary, *SlituOR3* is contributed to mediate the olfactory responses to Z9E11-14:OAc and Z9E12-14:OAc, and is related to the individual variation of *S*. *litura* olfactory system.
